# Encountering Meckel's diverticulum in emergency surgery for ascaridial intestinal obstruction

**DOI:** 10.1186/1749-7922-5-15

**Published:** 2010-06-09

**Authors:** Imtiaz Wani, Viliam Šnábel, Ghulam Naikoo, Shadab Wani, Muddasir Wani, Abid Amin, Tariq Sheikh, Fazal Q Parray, Rauf A Wani

**Affiliations:** 1 Department of Surgery, SMHS Hospital, Srinagar, Kashmir, India; 2 Parasitological Institute, Slovak Academy of Sciences, Košice, Slovakia; 3 Department of Pediatrics, GB Pant Hospital, Srinagar, Kashmir, India; 4 Department of Surgery, SKIMS, Srinagar, Kashmir, India

## Abstract

**Background:**

Meckel's diverticulum is the most common congenital anomaly of the gastrointestinal tract. In children with intestinal ascariasis, the diverticulum remains asymptomatic or rarely the *Ascaris lumbricoides *may lead to its complications in the presence of massive intestinal roundworm load. Given that preoperative diagnosis is seldom carried out, when Meckel's diverticulum is found at laparotomy for obstructive intestinal complications of roundworm, the diverticulum should be removed as complications may occur at any time. The aim of this study was to describe the findings of concomitant presence of Meckel's diverticulum who had surgical intervention in symptomatic intestinal ascariasis in children.

**Methods:**

A retrospective case review study of 14 children who had surgical intervention for symptomatic intestinal ascariasis having the presence of concomitant Meckel's diverticulum was done. The study was done at SMHS Hospital Srinagar, Kashmir.

**Results:**

A total of the 14 children who had ascaridial intestinal obstruction with concomitant presence of Meckel's diverticulum were studied. Age of children ranged from 4-12 years, male:female ratio was 1.8:1. Nine patients had asymptomatic Meckel's diverticulum, whereas 5 patients with symptomatic signs were found in the course of emergency surgery for ascaridial intestinal obstruction.

**Conclusion:**

Meckel's diverticulum in intestinal ascariasis may pursue silent course or may be accompanied with complications of the diverticulitis, perforation or the gangrene. Incidental finding of the Meckel's diverticulum in the intestinal ascariasis should have removal.

## Background

Though ascaris infestation is usually asymptomatic, ascariasis-related intestinal complications can be seen children with a high intestinal roundworm load. Presence of massive roundworm infestation in children may lead to symptomatic Meckel's diverticulum. High burden of intestinal roundworms, propensity to wander, size of the worm and the characteristics of Meckel's diverticulum constitute prerequisite for complications of Meckel's diverticulum. Surgical complications associated with *Ascaris lumbricoides *infection can be diverticulitis, gangrene or the perforation in the Meckel's diverticulum. Preoperative diagnosis of Meckel's diverticulum is often difficult. Incidental diverticulectomies in asymptomatic Meckel's diverticulum are considered safer [1,2]. The work was designed to study findings of concomitant Meckel's diverticulum who had surgical intervention for ascaridial intestinal obstruction in children.

## Methods

A retrospective case review study of 14 children who had surgical intervention for symptomatic ascaridial intestinal obstruction with the presence of the concomitant Meckel's diverticulum, was done at SMHS Hospital, Srinagar from March 1997-March 2009. All children were local ethnic population of Kashmir. Detailed clinical history and examination, abdominal X-ray and the ultrasonography abdomen were used for diagnosis.

## Results

A total of 14 patients having the presence of concomitant Meckel's diverticulum who had surgical intervention for ascaridial intestinal obstruction were encountered. No preoperative diagnosis of Meckel's diverticulum was made. Out of 14 children, 9 were male children and 5 were female children, youngest child was a 4 years old boy and oldest child was 12 years old girl child. Intestinal obstruction was present in 11 patients who did not respond to conservative management. Clinical features of the peritonitis were present in 3 patients. Size of Meckel's diverticulum ranged from 2 to 7.5 centimeter and diameter from 0.5 cm to 4.5 cm. All had location of Meckel's diverticulum at distance of 60 -80 centimeters from illeocaecal junction. Three cases had complications of Meckel's diverticulum secondary to roundworm induced complications (Gangrene) of ileum segment bearing Meckel's diverticulum due to worm boluses, one was a male child and the two were female children. Two cases had direct complications (diverticulitis, diverticulitis with perforation) of Meckel's diverticulum by roundworms, both cases were a male children. Nine patients had an incidental finding of grossly as well as histologically documented normal Meckel's diverticulum.

**Figure 1:**
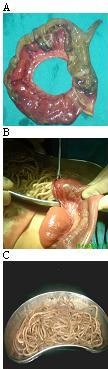
**Demonstration of ileum with its located Meckel's diverticulum, both had gangrene**. Ileum had twist which lead to gangrene of ileum, together with its located Meckel's diverticulum with worms seen inside. There was proximal and distal bolus of worms at point of twist around which ileum had volvulus. **B**. Demonstration of resected ends of ileum which had gangrene. Both resected ends were used as enterotomy sites for removal of worms. **C**. Demonstration of worms removed via enterotomy wound.

Three patients had gangrenous Meckel's diverticulum; one had secondary to volvulus of ileum caused by presence of worm bolus at proximal and distal end leading to gangrene of ileum and its located Meckel's diverticulum (Fig. 1A, B &1C). Two had secondary to mechanical obstruction to gut by long proximal worm bolus leading to gangrene of distal ileum with its associated Meckel's diverticulum.

**Figure 2:**
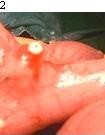
**Perforation at tip of Meckel's diverticulum through which worms escape into peritoneal cavity**.

One patient had markedly inflamed Meckel's diverticulum with single impacted roundworm present inside. Perforation of Meckel's diverticulum (Diverticulitis) with three roundworms present in peritoneal cavity was seen in one case (Fig 2). Two roundworms were wrapped in omentum and one was lying freely in peritoneal cavity.

Diverticulectomy was done in 9 cases and the segmental resection in 5 cases including resection anastomosis those who had gangrene of ileum. There was no presence of any ectopic tissue in specimens of Meckel's diverticulum on histopathology. Three patients had post operative wound infection. All were treated with anthelmintics postoperatively.

## Discussion

Meckel's diverticulum is the most common congenital anomaly of the gastrointestinal tract [3]. The occurrence of symptomatic Meckel's diverticulum in male and female has ratio of 3:1, with complications being more frequently encountered in males [4]. Reports from autopsy and retrospective studies show incidence ranges from 0.14 to 4.5%, 4.2% of cases were asymptomatic in a study from the U.S. [5].

**Figure 3:**
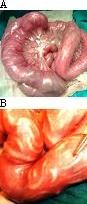
**A & B Showing of multiple long worm boluses present in small gut**.

**Figure 4:**
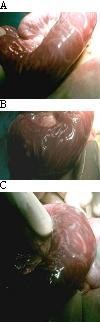
**A & B Showing of impacted long worm bolus with transerosal visibility**. **C**. Showing of impacted worm bolus with gangrene of distal small gut due to mechanical obstruction.

A variety of surgical complications in an abdomen caused by *Ascaris lumbrocoides *may arise and usually occur in the children. Wandering nature of *Ascaris lumbricoides *after migration from their usual habitat of small intestine leads to myriad of surgical complications in the abdomen. Intestinal obstruction, biliary ascariasis, pancreatic ascariasis, hepatic abscess, gallbladder ascariasis, hepatolithiasis, appendicitis and Meckel's diverticulitis are complications associated with the abdominal ascariasis. Among them, ascaridial intestinal obstruction is the most common complication seen in the children [6]. Mode of intestinal obstruction involves mechanical obstruction, intussusception or volvulus of small gut. Mechanical obstruction is the most frequent mode of small gut obstruction and is due to bolus of worms (Fig 3A, B & Fig 4A, B, C). Ascaridial intestinal obstruction can be manifested as partial or the complete type of small gut obstruction. In children, abdominal pain, vomiting and abdominal distension are usually present. There can be diarrhea, constipation, passage of worms with stools as well as with vomitus.

Management of intestinal ascariasis may involve conservative treatment or the surgical intervention to patients who do not respond to the conservative management. Plain X-ray abdomen and the ultrasonography abdomen are routinely used radiological investigations used for diagnosis. Conservative treatment implemented by application of intravenous fluids for hydration, antibiotics and use of enemas. Antihelminthics are given when patients are asymptomatic.

When deciding for for surgical intervention in ascaridial intestinal obstruction, **Wani criteria **[7] were used, and are as follows:

• Unsatisfactory response to conservative management

• Toxemia out of proportion to the severity of obstruction

• Increasing abdominal distension, guarding, and rebound tenderness

• Persisting abdominal pain and the tender worm mass

• Persistence of worm mass at the same site or fixity of mass

• Bleeding P/R in addition to above signs and symptoms

• Increasing distension of gut loops and number of free fluid levels or any evidence of volvulus or intussusception and the presence free gas under diaphragm suggestive of gut perforation on X-ray abdomen

• Ultrasonographic evidence of significant and progressively increasing interloop fluid or free fluid in peritoneal cavity and any evidence of peritonitis.

**Figure 5:**
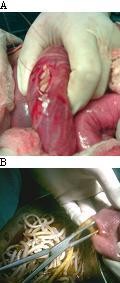
**Showing of enterotomy wound made after placing stay sutures for impacted long worm bolus with transerosal visbility**. **B **Showing diverticulectomy wound that was used as an enterotomy site for removal of worms.

Surgical interventions used in the ascaridial intestinal obstruction are enterotomy, milking and the resection anstomosis. The enterotomy to remove worms is based on opening the small gut wall through which worms are removed (Fig. 5A). Milking or kneading of worms involves manual pushing of worms into large colon where from they pass freely through rectum as roundworms do not cause large gut obstruction. Enterotomy is ranked as the most common surgical procedure that need surgical intervention due to ascaridial intestinal obstruction in children [7,8]. Enterotomy for removal of roundworms is usually done in cases with impacted worm boluses with transerosal visibility or if the worms cannot be milked down into the colon. The presence of long impacted worm bolus or small boluses with transerosal visibility requires enterotomy to remove worms given the risk of serosal tears of small gut if milking of worms is attempted in such cases. Sometimes multiple enterotomies are to be done when multiple impacted worm boluses widely apart in small gut are present.

Peroperative findings in these series favoured enterotomy as a main surgical procedure; patients who had gangrene of small bowel had undergone resection. Resected ends of small bowel were used as enterotomy site for removal of worms in those who had segmental resection for Meckel's diverticulum or who had gangrene of small gut (Fig. 1B). Kneading of worms towards resected ends after enterotomy ensures complete removal of round worms from small gut, if particularly small parasites are left. In this series, in patients with incidental finding of asymptomatic Meckel's diverticulum during surgeries, diverticulectomy was done in all cases and the same wound was used as an enterotomy site for removal of worms.(Fig. 5B).

Association of *Ascaris lumbricoides *with Meckel's diverticulum in children only rarely leads to its complications. In areas where *Ascaris *infestation is endemic, heavy worm infestation may lead to Meckel's diverticulitis secondary to incarceration of round worm in a Meckel's diverticulum [9]. Number of individual migrating worms is low as they usually remain as entangled masses in ileum and thus incarceration is seldom seen. Worms can transiently stay and then migrate out of Meckel's diverticulum due to its wandering nature, self-emptying characteristic of Meckel's diverticulum and the presence of peristalsis by virtue of smooth muscle in the wall of this diverticulum. Incarceration is usually caused by small sized roundworm in the long diverticulum with relatively narrow diameter where round worms have a possibility during curling movements to undergo incarceration by knotting or by getting impacted in diverticulum (this was seen in one case).

Gangrene of Meckel's diverticulum has been linked with intake of iron tablet in pregnancy, persistent omphalomesentric duct, axial torsion and in strangulated hernia [10,11]. Sometimes gangrene of Meckel's diverticulum occurs in an ascaridial intestinal obstruction following volvulus of ileum segment, with its located diverticulum due to worm bolus (Fig. 1A). Direction of volvulus is usually clockwise direction. Proximal worm bolus induced mechanical obstruction can occasionally lead to the gangrene of ileum and its located Meckel's diverticulum.

Perforation of Meckel's diverticulum is rarely seen implied by the roundworms, fishbone, iron nail, drugs, spontaneous, toothpick and the button hole battery [12-14]. *Ascaris lumbricoides *is able to perforate Meckel's diverticulum and can lead to the panperitonitis [15-18]. Under the presence of pathological Meckel's diverticulum with perforation can roundworms sometimes migrate through this route into peritoneal cavity. Perforation is usually seen at the tip of inflamed diverticulum. Pressure necrosis from the impacted worm and oedema around the neck of the diverticulum may lead to narrowing of the opening in pathological Meckel's diverticulum and impeding vascular supply that probably resulted in these perforations. It should be stressed that worm itself directly cannot lead to perforation of normal Meckel's diverticulum.

In justifying prophylactic removal of silent Meckel's diverticulum in course of emergency surgical intervention for obstructive ascaridial intestinal obstruction is supported by observations that diverticulectomy or resection of Meckel's diverticulum do not likely incur a significant amount of postoperative morbidity due to postoperative intestinal obstruction, and infection or the rate of complications from a diverticulectomy are low [19,20]. Moreover, the use of diverticulectomy wound as an enterotomy site for complete removal of worms, favors incidental diverticulectomy in course of surgery of ascaridial intestinal obstruction.

Wandering nature of *Ascaris lumbricoides *coupled with stress of surgical intervention stimulating propensity to migrate lead to panicky movements of worm to seek orifices for escape that may lead to postoperative complications if migrating in silent Meckel's diverticulum, if left in situ. Furthermore, while being worms removed via enterotomy wound or the milking of worms, there is a possibility of roundworm being iatrogenically lodged in the silent Meckel's diverticulum if left in situ that may cause postoperative complications.

## Conclusion

Meckel's diverticulum with intestinal ascariasis may remain asymptomatic or present with complications. *Ascaris lumbrocoides *can lead to direct complications of Meckel's diverticulum or secondarily after having complications of ileal segment on which it is located. Preoperative diagnosis is difficult. Silent Meckel's diverticulum encountered during the course of surgery for obstructive intestinal ascariasis in children is to be removed in view of anticipated complications. Diverticulectomy wound can be used as enterotomy site for complete removal of intestinal worms.

## Competing interests

The authors declare that they have no competing interests.

## Authors' contributions

IW, VS and GN prepared, analysed and revised final manuscript. SW, MM, AA, TS, FP and RW helped in final revision. All authors have read and approved the final manuscript.
